# The effects of different surgical approaches on the psychological status, medical coping mode and quality of life of patients with lung cancer

**DOI:** 10.3389/fpsyg.2023.1039501

**Published:** 2023-03-27

**Authors:** Yi-ping Chen, Yi Zhang, Xing Chen, Jiang Luo, Zhangqun Chen, Liping Zhao, Guili Xia, Xueqi Sui, Yunchen Li

**Affiliations:** ^1^Department of Gastroenterology, Shenzhen Hospital, Southern Medical University, Shenzhen, Guangdong, China; ^2^Hunan Key Laboratory of Oral Health Research & Xiangya Stomatological Hospital & Xiangya School of Stomatology, Central South University, Changsha, Hunan, China; ^3^Department of Nursing Research, Affiliated Hospital of Guangdong Medical University, Zhanjiang, Guangdong, China; ^4^Clinical Nursing Teaching and Research Section, The Second Xiangya Hospital of Central South University, Changsha, Hunan, China; ^5^Department of Pediatrics, The Second Xiangya Hospital of Central South University, Changsha, Hunan, China

**Keywords:** anxiety, depression, medical coping mode, psychological status, lung cancer, surgery

## Abstract

**Objective:**

This study aimed to compare the effects of robot-assisted thoracic surgery (RATS), video-assisted thoracic surgery (VATS), and thoracotomy on the psychological status, medical coping mode, and quality of life of patients with lung cancer.

**Methods:**

A total of 158 patients with lung cancer were selected from the thoracic surgery center of a third-grade hospital in Hunan Province, China, from September to November 2020. The Self-Rating Anxiety Scale (SAS), Self-Rating Depression Scale (SDS), Medical Coping Modes Questionnaire (MCMQ), and Medical Outcomes Study (MOS) 36-item Short Form Health Survey (SF-36) were used to assess the effects of the surgical approaches on the study parameters before and 48–96 h after surgery. The t-test and analysis of variance were used to analyze the data.

**Results:**

The results revealed that the patients’ depression increased, their short-term quality of life decreased, and they tended to adopt a positive coping mode after surgery (*p* < 0.05). The RATS and VATS groups differed in avoidance dimension of medical coping modes (*p* < 0.05). The VATS and thoracotomy groups differed in the body pain dimension of quality of life (*p* < 0.05). Different surgical approaches had no effect on the psychological status, medical coping modes except the avoidance dimension, and quality of life except the body pain dimension.

**Conclusion:**

Surgical approaches have little effect on the psychological status, medical coping modes, and quality of life of patients with lung cancer; however, their depression increased and quality of life decreased after the surgery.

## 1. Introduction

Lung cancer is one of the prevalent malignant tumors and major causes of death worldwide (Patricia et al., 2018). Surgical resection with curative intent remains the gold standard for treating patients with lung cancer. (Deffebach and Humphrey, 2015; Ettinger et al., 2022). Surgery is the preferred treatment of choice for stage I, stage II, and some stage III non-small cell lung cancers and stage I and some stage IIA small cell lung cancers. It can effectively remove diseased tissues and improve patient survival rates (Zhi et al., 2015; Suzuki et al., 2019; Saji et al., 2022). Current surgical approaches include robot-assisted thoracic surgery (RATS), video-assisted thoracic surgery (VATS), and thoracotomy, and each has specific advantages and disadvantages. Thoracotomy has a short operation time, but involves large incision and trauma, and difficulty in recovery. VATS has the advantages of less trauma and easy recovery, but involves a long and complex operation (Wang et al., 2015; Huang et al., 2016). Compared with VATS, RATS has a shorter hospital stay, less bleeding, more radical lymph node dissection, and more accurate surgical incisions (Louie et al., 2012; Farivar et al., 2014; Wilson et al., 2014).

Studies have reported a high incidence of anxiety and depression (33–44%) in patients with lung cancer during diagnosis and treatment (Shimizu et al., 2012; Gu et al., 2018). Moreover, patients may experience psychological problems such as anger, feelings of isolation, anxiety, depression, and lower self-esteem after surgery. Coping is the process through which a person adopts different strategies to deal with a problem (Darabos et al., 2021). Patients with lung cancer adopt a more positive coping mode after the surgery compared with that in the preoperative stage; however, the specific medical coping modes corresponding to different surgical methods are unknown. Perioperative quality of life is an important factor and prognostic indicator of the survival and recovery of patients with lung cancer (Morrison et al., 2017; Mederos et al., 2020; Zheng et al., 2020), and it is also the ultimate goal of patient treatment (Buitron et al., 2018). Previous studies have shown that the quality of life of patients with lung cancer was associated with their depression and anxiety (Jung et al., 2018; Polański et al., 2018).

Existing studies have focused on comparing the effects of the three surgical approaches on surgical complications and oncological outcomes for patients regarding survival or recurrence after the surgical treatment (Jiang et al., 2019; Meng, 2020). Psychological status and quality of life after surgery are equally or even more important for patients. However, previous studies have shown no statistically significant difference in quality of life of patients with lung cancer in the thoracotomy and VATS groups (Hopkins et al., 2017). With increasing medical innovation in the field of surgical intervention, new evidence is needed to confirm the reliability of the results. Accordingly, we aimed to compare the effects of surgical approaches on the psychological status, medical coping mode, and quality of life of patients with lung cancer, providing a basis for prevention and intervention to help the patients improve their psychological health and quality of life.

## 2. Materials and methods

### 2.1. Patient enrollment

We included all patients with lung cancer who underwent surgery at the thoracic surgery center of a hospital in Hunan Province, China, from September to November 2020.

The inclusion criteria were as follows: (a) diagnosed with lung cancer, (b) underwent surgery, and (c) volunteered for the study. Correspondingly, the exclusion criteria were: (a) inadequate literacy level, (b) severe mental disorders, and (c) inadequate communication ability.

The ethics approval was obtained from the ethics committee of Central South University [No: E2020178]. Written informed consent to participate in this study was provided by the participants.

### 2.2. Measures

Health-related quality of life was measured using the Medical Outcomes Study (MOS) 36-item Short Form Health Survey (SF-36) compiled by Stewart et al. (1988). The scale comprises 36 items and eight dimensions, including physical function, role-physical function, and body pain. The scores for the SF-36 were converted to a range from 0 to 100 using the formula (*raw sum – possible minimum score*) ÷ (*possible maximum score – possible minimum score*). This scale is one of the commonly used health measurement tools worldwide. The Cronbach’s alpha for the SF-36 was 0.899.

Anxiety was determined using the Self-Rating Anxiety Scale (SAS) compiled by Zung (1971). The scale consists of 20 items, and each item is ranked from 1 to 4, with higher scores indicating higher levels of anxiety. According to the Chinese norm (Dai, 2010), the criteria for severity were as follows: no anxiety (score < 50), mild anxiety (score 50–59), moderate anxiety (score 60–68) and severe anxiety (score ≥ 69). The Cronbach’s alpha for the SAS was 0.864.

Depression was determined using the Self-Rating Depression Scale (SDS) compiled by Zung (1965). The scale consists of 20 items, and each item is ranked from 1 to 4, with higher scores indicating higher levels of depression. The criteria for severity were as follows: no depression (score < 53), mild depression (score 53–62), moderate depression (score 63–72), and severe depression (score ≥ 73) (18). The Cronbach’s alpha for the SDS was 0.873.

The coping mode was measured using the Medical Coping Modes Questionnaire (MCMQ) compiled by Feifel et al. (1987) and translated into Chinese by Shen (2000). The scale comprises 20 items and three dimensions: confrontation (8 items), avoidance (7 items), and acceptance-resignation (5 items). Each item is ranked from 1 to 4. The dimension with the highest cumulative score indicated the particular coping strategy that patients were most likely to use (Zeng et al., 2021). Cronbach’s alpha of the three dimensions were 0.69, 0.60, and 0.76, respectively.

### 2.3. Statistical analysis

Data were analyzed using SPSS 18.0. Descriptive data were summarized as frequencies and percentages. The scores were expressed as mean ± standard deviation (X ± S). Differences between groups were analyzed using the paired-sample t-test or analysis of variance, and *p*-values of less than 0.05 were considered statistically significant.

## 3. Results

### 3.1. Patient characteristics

In total, 158 patients enrolled in this study. The cohort consisted of 73 men (46.2%) and 85 women (53.8%); 11 (7.0%), 44 (27.8%), and 103 (65.2%) were < 30 years, 30–50 years, and > 50 years of age.

According to the surgical method, the participants were categorized in three groups, 24, 121, and 13 in the RATS, VATS, thoracotomy groups, respectively. As shown in Table 1, there was no statistically significant difference in gender, age, body mass index (BMI), psychological status, medical coping mode, and quality of life among the three groups (*p* > 0.05), and they were comparable.

**Table 1:** Comparison of general data of three groups of patients.

Variable		Groups			*χ*^2^/F	*p*
		RATS group (*n* = 24)	VATS group (*n* = 121)	Thoracotomy group (*n* = 13)		
Gender (*n*)	Male	12	52	9	3.42	0.181
	Famale	12	69	4		
Age (*y*)	≤30	0	10	1	3.606	0.462
	31 ~ 50	5	36	3		
	≥51	19	75	9		
BMI (kg /m^2^)		23.265 ± 2.578	23.278 ± 2.997	22.833 ± 2.900	0.138	0.827
Psychological status	anxiety	33.07 ± 10.432	33.50 ± 8.226	30.29 ± 8.987	0.812	0.446
	depression	28.70 ± 10.794	31.89 ± 11.852	27.12 ± 11.126	1.546	0.216
Medical coping mode	confrontation	17.50 ± 2.265	16.66 ± 2.340	17.23 ± 1.833	1.55	0.216
	avoidance	15.96 ± 3.071	15.01 ± 2.940	13.85 ± 2.940	2.217	0.112
	acceptance-resignation	8.33 ± 1.761	8.12 ± 1.212	8.08 ± 0.494	0.314	0.731
Quality of life	PF	90.63 ± 9.126	89.21 ± 13.292	86.92 ± 16.01	0.343	0.710
	RP	72.92 ± 38.951	74.79 ± 37.151	66.39 ± 43.94	0.367	0.694
	BP	75.46 ± 16.376	72.64 ± 17.036	81.20 ± 11.462	1.714	0.184
	GH	49.17 ± 19.318	47.52 ± 18.282	55.39 ± 13.457	1.13	0.326
	VT	72.50 ± 13.831	66.61 ± 17.586	72.31 ± 13.786	1.7	0.186
	SF	82.29 ± 25.780	85.64 ± 17.873	86.54 ± 17.277	0.338	0.713
	RE	72.22 ± 40.129	82.64 ± 33.637	92.31 ± 19.971	1.625	0.200
	MH	57.33 ± 18.971	56.83 ± 18.147	64.92 ± 16.261	1.173	0.312

### 3.2. Comparison of psychological status, medical coping modes, and quality of life before and after surgery

As shown in Table 2, surgery had no effect on the anxiety scores of patients with lung cancer (*p* > 0.05), however, it increased their depression scores (*p* < 0.05). In terms of medical coping modes, the score of the confrontation and acceptance-resignation dimensions increased, and that of avoidance dimension decreased after surgery (p < 0.05). Regarding quality of life, there was no statistically significant difference between the changes in general health and mental health scores at 48–96 h after surgery (*p* > 0.05), while the scores of other dimensions decreased (*p* < 0.05).

**Table 2:** Comparison of psychological status, medical coping mode and quality of life of lung cancer patients before and after surgery.

Variable		Before surgery	48 h-96 h after surgery	*t*/*F*	*p*
*Psychological status*					
	anxiety	33.17 ± 8.638	31.37 ± 9.890	1.864	0.064
	depression	31.01 ± 11.686	35.63 ± 10.805	−4.184	<0.001
*Medical coping mode*					
	confrontation	16.84 ± 2.302	17.41 ± 1.909	−2.433	0.016
	avoidance	15.06 ± 2.982	14.39 ± 2.516	2.313	0.022
	acceptance-resignation	8.15 ± 1.266	8.47 ± 1.250	−2.286	0.024
*Quality of life*					
	PF	89.24 ± 12.945	72.46 ± 18.345	8.864	<0.001
	RP	73.73 ± 37.835	15.51 ± 31.116	15.715	<0.001
	BP	73.77 ± 16.647	58.30 ± 17.898	8.591	<0.001
	GH	48.42 ± 18.130	43.16 ± 16.065	3.155	0.002
	VT	67.97 ± 16.883	49.87 ± 10.766	12.282	<0.001
	SF	85.21 ± 19.129	57.83 ± 21.742	12.057	<0.001
	RE	81.86 ± 33.984	60.55 ± 39.964	5.473	<0.001
	MH	57.57 ± 18.153	60.38 ± 17.594	−1.653	0.100

### 3.3. Comparison between the effects of surgical approaches on the psychological status, medical coping modes, and quality of life

As shown in Table 3, the avoidance dimension score of medical coping modes for the RATS group was higher than that for the VATS group (*p* < 0.05), and the body pain dimension score of quality of life for the thoracotomy group was higher than that for the VATS group (*p* < 0.05). Different surgical approaches had no effect on the total scores of psychological status, medical coping modes except for the avoidance dimension, and quality of life except for the body pain dimension of patients with lung cancer (*p* > 0.05).

**Table 3:** Comparison of the effects of different surgical approaches on the psychological status, medical coping modes and quality of life of patients with lung cancer.

Variable		Group			*F*	*p*
		RATS group (*n* = 24)	VATS group (*n* = 121)	Thoracotomy group (*n* = 13)		
psychological status	anxiety	29.53 ± 7.624	31.64 ± 10.060	32.21 ± 12.185	0.505	0.605
	depression	33.80 ± 8.881	36.30 ± 11.087	32.79 ± 11.275	1.027	0.360
medical coping mode	confrontation	16.83 ± 1.711	17.53 ± 1.928	17.38 ± 2.022	1.336	0.266
	avoidance	15.63 ± 2.961^*^	14.18 ± 2.306^*^	14.08 ± 3.040	3.516	0.032
	acceptance-resignation	8.17 ± 1.204	8.53 ± 1.285	8.46 ± 0.967	0.839	0.434
quality of life	physical function	16.67 ± 34.315	15.08 ± 30.014	17.31 ± 37.339	0.049	0.952
	role-physical	75.00 ± 14.295	71.41 ± 19.281	77.69 ± 15.494	0.958	0.386
	body pain	63.89 ± 20.264	56.29 ± 17.376^#^	66.67 ± 14.344^#^	3.458	0.034
	general health	45.42 ± 14.664	42.39 ± 16.035	46.15 ± 19.165	0.596	0.552
	vitality	53.13 ± 10.301	48.80 ± 10.993	53.85 ± 7.403	2.633	0.075
	social function	64.06 ± 20.626	55.79 ± 21.952	65.38 ± 19.199	2.346	0.099
	role emotional	75.00 ± 31.470	58.13 ± 41.394	56.41 ± 36.980	1.882	0.156
	mental health	65.00 ± 13.920	59.37 ± 18.389	61.23 ± 15.611	1.042	0.355

*RATS group vs VATS group, *p* < 0.05. #: VATS group vs thoracotomy group, *p* < 0.05.

## 4. Discussion

### 4.1. Patients’ depression scores increased and quality of life scores decreased post-surgery

Our study found an increase in patients’ postoperative depression scores and a decrease in quality of life scores compared to those in the pre-operative period. This is consistent with the study by Zhu et al. (2019) and may be related to the significant postoperative physical changes of patients (Jian et al., 2016). Depression is a common complication of surgical trauma and stress. Previous studies have indicated that patients with cancer complicated by depression have a 19% higher fatality rate than those with cancer alone (Pinquart and Duberstein, 2010). Moreover, long-term experience of depression and other mental disorders can activate the neuroendocrine immune regulatory network, leading to destruction of the body’s cellular immunity and resulting in a decline in the immune function of patients with cancer. This can induce tumors or lead to aggravation and deterioration of patients with tumor. In addition, negative emotions can easily cause patients not to cooperate with treatment or even to discontinue it. Patients may experience loss of appetite, sleep disorders, etc. which may reduce their quality of life (Wang et al., 2016). Li et al. (2014) formulated a group psychotherapy plan for patients with lung cancer. The main techniques used include psychoeducation, rational-emotional behavior therapy, support-expression, confidence therapy, painting, meditation relaxation training, and abdominal breathing training. The results of this study found that group psychotherapy based on psychoeducation and supportive expression could improve the social function and emotional state of patients and reduce depression in patients with lung cancer. Therefore, medical staff should pay attention to the depressive symptoms of patients with lung cancer after surgery and promptly detect and provide individualized psychological care to improve treatment compliance, quality of life, and achieve the best treatment effect.

### 4.2. Patients with lung cancer were more inclined to adopt positive coping modes after surgery

Among the three dimensions of the MCMQ, “confrontation” is regarded as a positive and effective coping mode, and “avoidance” and “acceptance-resignation” are regarded as negative coping modes. The results showed the participants’ confrontation and acceptance-resignation dimension scores increased, and the avoidance dimension score decreased. The score of the confrontation dimension was much higher than that of the avoidance and acceptance-resignation dimensions, which implies that patients tend to adopt a more active coping mode to confront diseases after surgery. Coping mode is an important intermediary variable of psychological stress. Individuals may adopt completely different coping modes when facing the same stress, which causes different psychological effects (Ju and Xu, 2019). Therefore, strengthening the education of coping modes and helping patients actively face surgery and illness are important ways to improve their quality of life. Wang (2019) found that the quality of life of patients with lung cancer was significantly related to social support and self-efficacy. Social support can improve the psychological status of patients with cancer by reducing negative emotions and regulating coping mechanisms (Li et al., 2016; Hajek et al., 2017). Self-efficacy is a predictor of patients’ ability to cope with the disease and adopt healthy behaviors. Self-efficacy affects health outcomes by influencing patients’ attitudes toward difficulties (Hellström et al., 2009). Yeung and Lu (2014) found that high levels of self-efficacy play an important role in optimizing the quality of life of patients with cancer. Therefore, medical staff can take relevant measures to enhance confidence and self-efficacy of patients to help them overcome the disease to improve their quality of life.

### 4.3. Different surgical methods have little effect on the patient’s psychological status and short-term quality of life after surgery

Our research showed that the RATS and VATS groups differed only in the avoidance dimension of medical coping modes, and that the VATS and thoracotomy groups differed in the body pain dimension of quality of life, which may be related to the different sizes of wounds caused by different surgical methods. Previous studies have shown that other factors affecting the mental health of patients with lung cancer include the type of lung cancer (Zeilinger et al., 2022), type of resection of the surgery (Hao et al., 2016), pathological stage (Yan et al., 2019), postoperative dyspnea, and severe pain (Morrison et al., 2017). There is moderate-quality evidence that preoperative exercise significantly reduces postoperative complication rates (Steffens et al., 2018). Therefore, it is essential that physicians make a reasonable preoperative assessment of these influencing factors, select a suitable surgical method, and assist patients with early protective rehabilitation programs, including pre-pulmonary surgery aerobic exercise and respiratory muscle training (Barğı et al., 2021). In addition, special attention should be paid to pain care of patients with major surgical wounds.

Different surgical methods have no effect on the psychological status, and little effect on medical coping modes (except avoidance dimension) and quality of life (except the body pain dimension) of patients with lung tumors, which suggests that the three surgical methods have equivalent effects on the short-term psychological status and quality of life after surgery. This highlights that the surgical method should be chosen based on the patient’s condition.

### 4.4. Research implications and clinical practice

This study revealed the changes in psychological status, medical coping modes, and quality of life of patients with lung cancer before and after surgery, as well as the differences in these parameters for the patients who underwent RATS, VATS, and thoracotomy. It provides a reference and theoretical basis for medical professionals to pay timely attention to the changes and formulate interventions for patients with lung cancer to improve their psychological status and quality of life after surgery.

According to the results of this study, the depression of patients increased and their short-term quality of life decreased after surgery. It is difficult for surgeons to evaluate patients’ psychological status, and the actual incidence of psychological distress may be underestimated. Therefore, patients should be carefully screened for psychological status by physicians using appropriate methods throughout the treatment process, and those with psychological distress should be managed by psychiatrists who can provide appropriate professional mental health care. In addition, our research showed that the different surgical methods had little effect on the psychological status, medical coping modes, and short-term quality of life of patients with lung cancer after surgery. This suggests that the surgical approach for patients with lung cancer is not universal and should be individualized; that is, the focus of surgery should be to make a holistic and comprehensive assessment to select the most appropriate procedure considering the patient’s physical condition. This reminds healthcare professionals of the need to pay attention to the psychological condition of patients when treating lung cancer and to use individualized treatment and care with the ultimate goal of improving the patient’s quality of life.

### 4.5. Study limitations

Our study has some limitations. First, it only focused on the impact of different surgical approaches on patients with lung cancer, and other factors such as types of lung cancer, pathological stage, postoperative dyspnea, severe pain, or comorbidities were not considered. This should be considered in future prospective cohort studies. Second, the formal sample size was not calculated beforehand, and we included all patients with lung cancer who met the selection criteria in a specific time period. It is necessary to expand the scope to include more patients. Finally, we did not follow up the discharged patients for a long time; therefore, a longer follow-up tis necessary to update the state of patients’ health in the future.

## 5. Conclusion

There is little difference in the effects of various surgical approaches on the psychological status, medical coping modes, and quality of life after 48–96 h of surgery of patients with lung cancer. However, their depression increased and quality of life decreased after surgery; therefore, it is necessary to focus on the psychological status of the patients and improve their quality of life after surgery.

## Data availability statement

The raw data supporting the conclusions of this article will be made available by the authors, without undue reservation.

## Ethics statement

The ethics approval was obtained from the ethics committee of Central South University [No: E2020178]. The patients/participants provided their written informed consent to participate in this study.

## Author contributions

YL and GX are the primary investigator of the study. Y-pC and YZ conducted the study, data analysis, and drafted the manuscript. XC, ZC, JL, LZ, GX, and XC helped conduct the study and proof reading the manuscript. All authors contributed to the article and approved the submitted version.

## Funding

This work was supported by Hunan Province Disabled Rehabilitation Scientific Research Project, China (fund number: 2019XK007) and Clinical Nursing and Management Perso-nnel Research Start-up Foundation of the Xiangya Stomatological Hospital of Central South University (No. HG202303).

## Conflict of interest

The authors declare that the research was conducted in the absence of any commercial or financial relationships that could be construed as a potential conflict of interest.

## Publisher’s note

All claims expressed in this article are solely those of the authors and do not necessarily represent those of their affiliated organizations, or those of the publisher, the editors and the reviewers. Any product that may be evaluated in this article, or claim that may be made by its manufacturer, is not guaranteed or endorsed by the publisher.
